# Extreme Environments and High-Level Bacterial Tellurite Resistance

**DOI:** 10.3390/microorganisms7120601

**Published:** 2019-11-22

**Authors:** Chris Maltman, Vladimir Yurkov

**Affiliations:** 1 Department of Biology, Slippery Rock University, Slippery Rock, PA 16001, USA; 2 Department of Microbiology, University of Manitoba, Winnipeg, MB R3T 2N2, Canada; vladimir.yurkov@umanitoba.ca

**Keywords:** tellurite, tellurite resistance, extreme environments, metalloids, bioremediation, biometallurgy

## Abstract

Bacteria have long been known to possess resistance to the highly toxic oxyanion tellurite, most commonly though reduction to elemental tellurium. However, the majority of research has focused on the impact of this compound on microbes, namely *E. coli*, which have a very low level of resistance. Very little has been done regarding bacteria on the other end of the spectrum, with three to four orders of magnitude greater resistance than *E. coli*. With more focus on ecologically-friendly methods of pollutant removal, the use of bacteria for tellurite remediation, and possibly recovery, further highlights the importance of better understanding the effect on microbes, and approaches for resistance/reduction. The goal of this review is to compile current research on bacterial tellurite resistance, with a focus on high-level resistance by bacteria inhabiting extreme environments.

## 1. Introduction

Microorganisms possess a wide range of extraordinary abilities, from the production of bioactive molecules [1] to resistance to and transformation of highly toxic compounds [2,3,4,5]. Of great interest are bacteria which can convert the deleterious oxyanion tellurite to elemental tellurium (Te) through reduction. Currently, research into bacterial interactions with tellurite has been lagging behind investigation of the oxyanions of other metals such as nickel (Ni), molybdenum (Mo), tungsten (W), iron (Fe), and cobalt (Co). This is mainly due to the fact that the latter metals have long been known as essential for life through their involvement in many cellular activities [6,7,8]. Bacterial interactions with tellurite have received attention [9,10,11,12], but much is still unknown about high-level resistance. In recent years, there has been more focus in this area due to increased environmental contamination from industrial activities [13,14]. Microbial reduction of tellurite, under both aerobic and anaerobic conditions, results in the detoxification of this harmful compound [14,15,16,17,18]. Therefore, tellurite reducers have an important role in nature. Their removal of this contaminant can allow many species to grow in environments with elevated toxic metal(loid) concentrations [14,17,18,19,20,21,22,23,24,25]. This review aims to summarize what is known in the area of high-level tellurite resistance by bacteria that call extreme habitats home.

### 1.1. Habitats of Highly Resistant Microbes

Extreme environments are habitats where one would not expect to find life. Conditions there represent the limits at which organisms can survive [18,19,20,21,22]. Hence, species diversity is low, and some major taxonomic groups are not present [26]. In order for microbes to exist in such abnormal areas, they must have undergone extensive adaptations, selected over time by nature. In fact, it has been found that evolution in extreme environments has been shown to be faster than in more moderate locales [27]. Microorganisms inhabiting harsh ecological niches that have optimal fitness only under such radical conditions are deemed extremophiles. Those that grow best at, or near to, conventionally ‘anthropocentric’ conditions, but can tolerate more extreme situations, are considered extremotolerant. They are classified based on the parameter which can be endured, such as pH, temperature, pressure, salinity, and water availability. Those able to withstand high levels of metal(loid)s have been referred to as metallophiles [28]; however, this description is somewhat misleading, as the suffix ‘phile’ implies better fitness in the presence of metal(loid)s, and this is generally not the case for tellurite, as even microbes with extreme levels of resistance do not necessarily grow better with such increased amounts, nor do they require the oxyanion to survive. Therefore, the term will not be used in this review. A more appropriate term would be ‘metallotolerant’.

Investigation of the microbial communities which have managed to establish themselves under harsh conditions has helped further our understanding of how life has evolved. Even though extreme environments have a low global distribution, they harbor a relatively high proportion of microbes considered valuable to science and technology [29], such as antibiotic-producing bacteria [30] and those used in the biodegradation of pollutants [31]. These habitats contain representatives from many genera, providing a wealth of information on biodiversity and microbial physiology. Furthermore, many of them possess the ability to resist and reduce very high levels of toxic metal(loid) compounds, specifically, tellurite.

### 1.2. Deep-Sea Hydrothermal Vents

**Figure 1:**
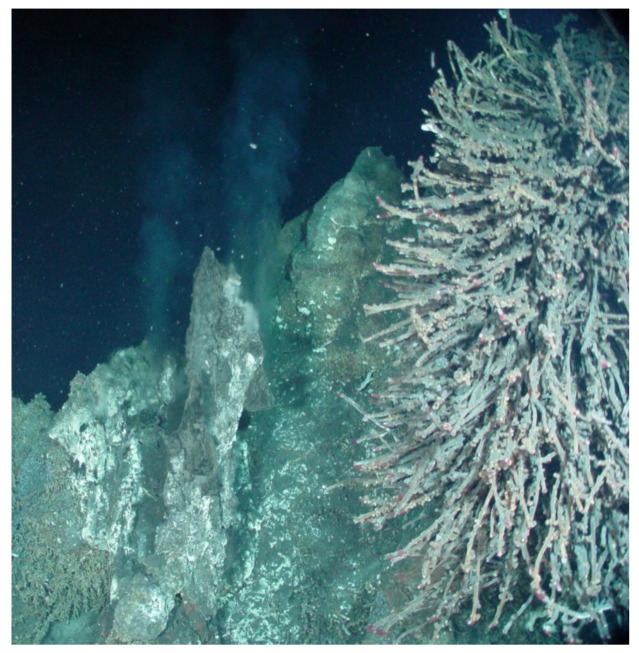
Photograph of deep-ocean hydrothermal vent and associated tube worms at the Juan de Fuca Ridge Vent Field in the Pacific Ocean. (Archives of V. Yurkov laboratory. Used with permission).

Considered to be the closest match to conditions present early in the Earth’s development, deep-sea hydrothermal vents are of great interest. However, our knowledge of these locales, including how common/rare they actually are, is limited, with new discoveries emerging from each expedition [32]. Much of this is due to logistical problems associated with exploring areas of the deep ocean. The vents themselves are only present in areas of geological instability in the ocean floor, such as faults and spreading centers, like the Juan de Fuca Ridge in the eastern Pacific Ocean [33]. They result from the expulsion of sea water which has percolated into the rock and sediment of the ocean floor and been superheated by subsurface magma (up to 400 °C) and acidified (pH as low as 3.3). The water is then enriched with H_2_S and various metal(loid)s which have been leached from the basalt comprising the ocean floor [34]. Once released into the cold conditions of the deep ocean (~2 °C), rapid cooling takes place, resulting in the dissolved minerals precipitating out of solution, appearing like a cloud of black smoke, hence the name ‘black smokers’. The process also leads to the deposition of metal-sulfides around the plume, giving the appearance of a chimney (Figure 1). Under the extreme pressure (250 ATM) and temperatures, Te can replace S in the chimney walls [35], thereby increasing the concentration of the element. These unique locations represent several extremes: pressure, temperature, acidity, and increased levels of tellurite. This last feature is ideal for directing microbial evolution towards the utilization of the usually-toxic compound for biological processes, or at the very least, the ability to resist and/or reduce it. Even though conditions at these sites would suggest a scarcity of life, many unique organisms have made this ecological niche their home [36]. Of particular interest are the sulfide and tube worms inhabiting vent systems (Figure 1). As a consequence of their proximity to plume waters, they are exposed to increased levels of tellurite, and therefore, so are their associated microbes [36]. As a result, these animals harbor a community of tellurite-resistant bacteria [18,22,37,38], indicating that the microbial population has adapted to exposure. Black smokers have provided us with examples of bacteria possessing very high levels of tellurite resistance [20,39,40]. Vents also supply chemicals, including Te, that are sufficiently reduced for energetically-expensive biochemical reactions [34], which led to the observation that this element can be used for survival and growth [18,22,41]. This all confirms that the environment provides conditions conducive to the evolution of resistance and biological reactions which are dependent on tellurite.

### 1.3. Thermal Springs

**Figure 2:**
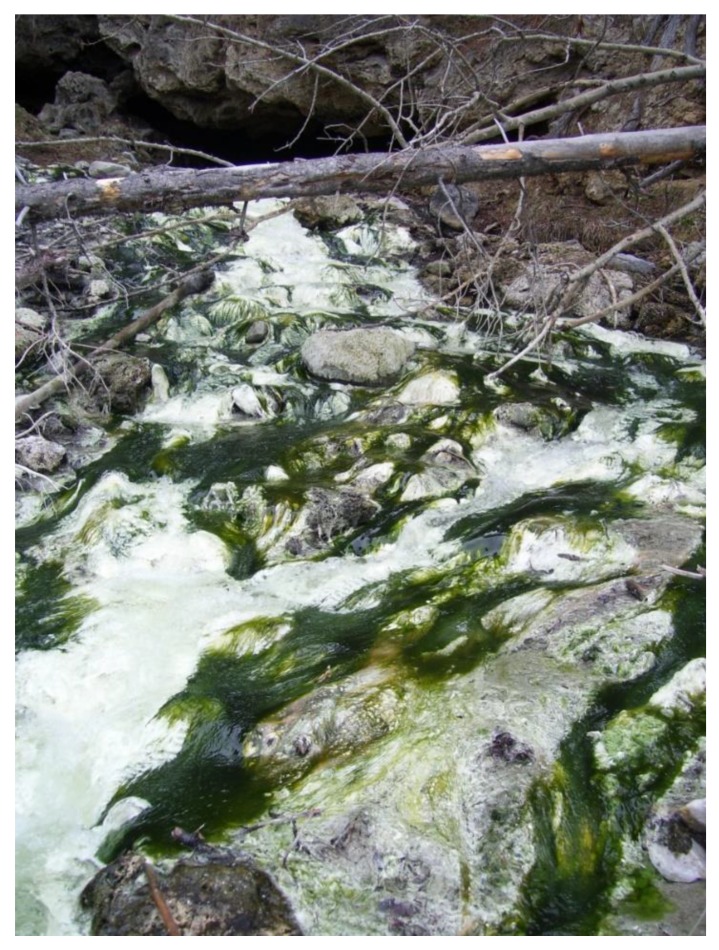
Sulfur Mountain thermal springs, Banff, Alberta, Canada. Outflow temperature is approximately 45 °C. The microbial mat forming community is comprised primarily of *Spirulina* (green) and *Thiothrix* like (white) microorganisms. (Archives of V. Yurkov laboratory. Used with permission).

Similar to deep-sea hydrothermal vents, thermal springs are produced by the emergence of geothermally-heated groundwater from the Earth’s crust (Figure 2). They range from small ground seepage to flowing rivers and, if under pressure, fountains or geysers. They are found at many different locations globally, and have a nearly ubiquitous association with faults and active hydrothermal outflow [42]. Since these sites of geothermal heating vary in temperature, so does the water that is expelled. Therefore, it has been under debate as to what water temperatures actually constitute a thermal spring. One definition states that the temperature must be greater than body temperature (36.7 °C), but it has been criticized as being anthropocentric in nature and neglecting to consider the surrounding environment. Another suggests higher than the mean ambient air temperature [43]; however, there is a problem with that definition. At locales of higher altitude and/or latitude, where temperatures never rise above 0 °C, water may be flowing at or near to freezing. Here, it would not be reasonable to consider the springs ‘thermal’. In the context of this review, the first definition has been adopted.

Water emerging from these springs passes through bedrock before it is ejected at the surface, leading to enrichment in leached minerals, much like the waters expelled by deep-sea hydrothermal vents. As a result, they are usually linked to increased concentrations of various metal(loid)s [44,45,46,47] and have provided examples of highly resistant bacteria [48,49,50]. Investigation of samples taken from the hot temperature springs of the Bolshaya river basin, Baykal Lake region, and Kamchatka Island in Russia led to the discovery of freshwater aerobic anoxygenic phototrophic bacteria (AAP) capable of resisting and reducing very high levels of tellurite [2,51,52,53,54,55]. Discoveries like this further highlight the importance of environments such as these as a source of highly resistant microbes.

### 1.4. Hypersaline Environments

**Figure 3:**
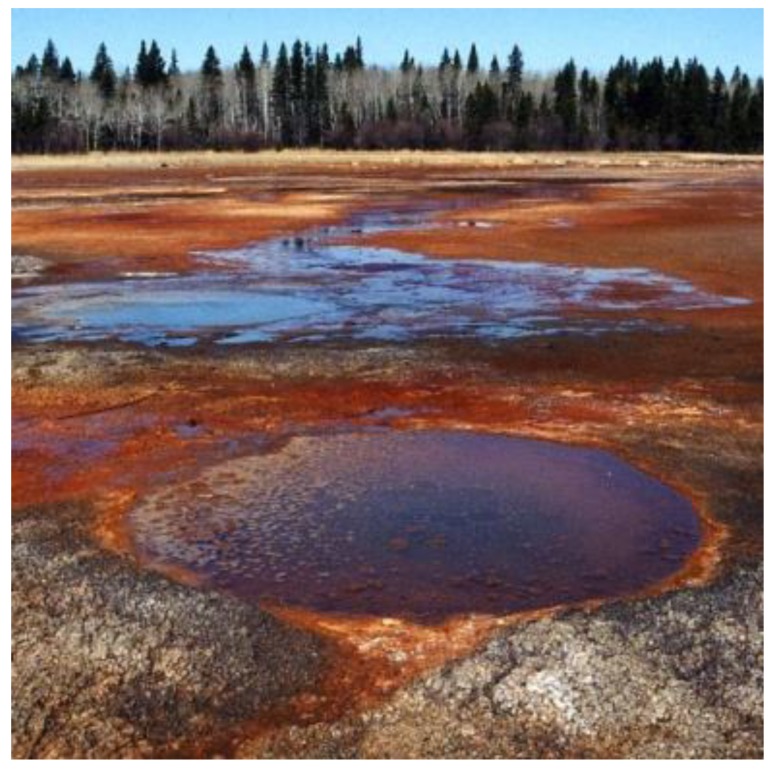
East German Creek hypersaline system, Manitoba, Canada. (Archives of V. Yurkov laboratory. Used with permission).

Hypersaline environments, where salinity exceeds marine values by about 3% [56], fall into three broad categories: (1) supralittoral marine pools and flats [57,58], (2) saline and meromictic lakes [59,60], and (3) hypersaline springs [61]. Salt-rich ancient marine sediments now exposed at the surface can enrich overlying lakes, sometimes causing them to accumulate sufficient solutes at depth to resist turnover of the hypolimnion, in which case they are called meromictic lakes [62]. Examples include the Great Salt Lake in the United States, the Dead Sea in Israel/Jordan, and Mahoney Lake in Canada [60,62]. Similarly, ground water passing through marine geological horizons can generate hypersaline springs (Figure 3). Compared to meromictic lakes and intertidal zones, hypersaline springs are relatively rare, because the conditions that need to be met for their existence are rather restrictive, i.e., sufficient hydrostatic pressure to force free-flowing subterranean water to the surface after passing through enough salt-rich strata to appreciably load it with solutes. 

The hydrology of hypersaline systems also elevates concentrations of metal(loid)s, including Te, above background levels [34,62,63,64]. Trace elements are concentrated during the evaporation of runoff from precipitation, irrigation, or spring water, leading to hypersaline pools with toxic levels of metal(loid)s [63]. The microorganisms residing in these locations are extensively adapted to regulating intra- and extra- cellular ion concentrations, leading to a preadaptation to dealing with concentrated metal(loid) oxyanions. In fact, hypersaline systems boast the highest microbial tellurite resistance ever recorded. The haloalkaliphilic archaea *Natronococcus occultus*, *Natronobacterium magadii*, and *Natronobacterium gregoryi* have a minimum inhibitory concentration (MIC) of 10 to 20 mM (2570 to 5140 μg/mL) for tellurite [65]. This is only possible because of the high solubility of tellurite in alkaline media. The hypersaline Mono Lake in California is also the source of the tellurite-respiring halophile *Bacillus beveridgei* (optimum 0.5 to 1.5 M NaCl, with growth up to 4 M) [66]. 

### 1.5. Mine Tailings

**Figure 4:**
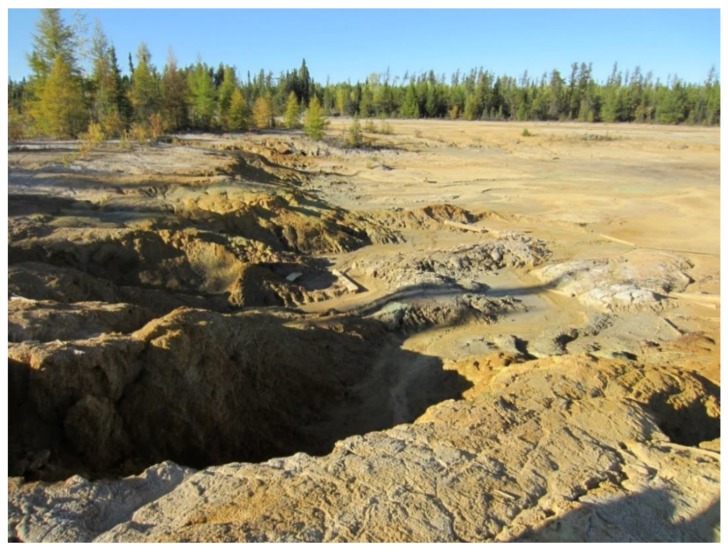
Gold mine tailings of the Central Mine, Nopiming Provincial Park, Manitoba, Canada. (Archives of V. Yurkov laboratory. Used with permission).

The mechanical and chemical methods used in mining to obtain the desired product from ore produce waste, primarily ground rock and process effluents, creating tailings. The extraction process is neither 100% efficient nor is it ever possible to reclaim all reusable and expended processing reagents and chemicals. The unrecoverable and uneconomic metals, minerals, chemicals, organics, and water are then discharged into the surrounding area (Figure 4). Te is often found in the ores of other desirable metals. During extraction of coal, gold, silver, copper, and various others, it can be released as a byproduct [67,68,69,70,71,72]. Therefore, effluent from mining operations is enriched in this element and its oxyanion, tellurite, leading to an environment that exerts selective pressure on the microbial community to evolve survival mechanisms. This habitat, much like deep-sea hydrothermal vents and thermal/salt springs, has provided pure cultures of bacteria showing very high-level resistance to tellurite [14,25,69,73].

### 1.6. Diversity of Tellurite-Resistant Microbes Inhabiting Extreme Environments

Microbial life can be found almost everywhere, no matter how severe the conditions [74]. From the deepest seafloor to the top of the highest mountain, or the hottest desert to the coldest Antarctic plain, microorganisms have been recovered. Extreme habitats can support diverse bacterial communities [18,75,76,77,78,79,80]. The investigation of the microbial communities which have managed to establish themselves under harsh conditions has helped further our understanding of how life evolved. Even though extreme environments have a relatively low global distribution, they harbor a relatively high proportion of microbes considered valuable to science and technology [29,30,31]. These habitats contain representatives from many genera, providing a wealth of information on biodiversity and microbial physiology. Furthermore, many of them possess the ability to resist and reduce very high levels of toxic metal(loid) compounds, such as tellurite. While many genera are represented across different locations, diversity at individual sites can be limited. For example, acid mine drainage is usually dominated by iron-oxidizing, acidophilic genera, such as *Ferrovum* or *Acidithiobacillus* [81,82], while the high-temperature and acidic environment at Yellowstone National Park, USA, gives rise to an endolithic community primarily comprised of *Mycobacterium* spp. [83]. With metal-contaminated locales, such as mine tailings or industrial effluent, microbial communities greatly differ from site to site [24,84,85,86]. Research into the microbial community composition of specific metalloid-enriched locales in or around hydrothermal vents has been previously undertaken, with a focus on low-temperature, diffuse-flow, deep-sea vents [87], vent plume waters [77], hydrothermal sediments [76], and vent chimney microbial mats [75,88,89]. These studies have shown that there can be extreme differences in the bacterial populations between neighboring vents. Some attention has also been given to the metabolic diversity [90], as well as vent worm epibionts [18,37,91]. *Riftia pachyptila* possesses endosymbiotic bacteria which are chemolithoautotrophic, sulfur-oxidizing endosymbionts, and autotrophically fix carbon dioxide, using reduced sulfur compounds in the vent fluids as electron donors. This action aids in cleaning the blood from toxic sulfide, and synthesizing organic compounds for their host [36]. However, the presence diversity of metal(loid) oxyanion-resistant bacteria associated with vent worms has only recently been considered. As these epibionts can remove toxic metal(loid)s dissolved in the surrounding water, they may have a role to play in detoxifying the blood of their hosts. Maltman et al. showed that the epibiotic microbial community of tube worms at the Axial Volcano (AV) and Explorer Ridge (ER) sites of the Juan de Fuca vent field in the Pacific Ocean was not only capable of resistance to tellurite, but that it was highly diverse [18]. The vent worm epibionts at AV were dominated by *Vibrio* (41.9%) and *Pseudoalteromonas* (39.5%) relatives, with *Curvibacter* (9.3%) and *Shewanella* (9.3%) relatives making up the remainder. However, the associated microbes of the worms at ER differed from AV. These isolates had increased variety, dominated by *Curvibacter* (36.5%) and *Shewanella* (30.2%) relatives. The remaining community were comprised of *Pseudomonas* (12.7%), *Pseudoalteromonas* (7.9%), *Marinobacter* (3.2%), *Thalassospira* (3.2%), *Vibrio* (3.2%), *Aquabacterium* (1.6%), and *Okibacterium* (1.6%) relatives. Lastly, there was also a great variety in sequence similarities to known species (from as low as 90.6% to as high as 100%) suggesting a highly-diverse microbial population at each of these locations. It appears that resistance to tellurite is not limited to one, or a small group of bacteria, but is a wide-spread ability which is present across various genera and species.

## 2. Tellurium and Tellurite

### 2.1. Chemistry and Abundance

Tellurium is a metalloid element related to oxygen and sulfur in group 16 of the periodic table. It possesses stable oxidation states of VI (tellurate, TeO_4_^2−^), IV (tellurite, TeO_3_^2−^), 0 (elemental tellurium, Te^0^), and II (telluride, Te^2−^). Overall, Te has a very low global abundance (10^−2^ to 10^−8^ ppm), and its distribution is not homogenous [67,92]. As a result of such low natural concentrations, it has garnered little attention with regards to its effect on microbes [93,94]. However, levels can be elevated in certain locales. In gold mines, it can be significantly concentrated (14.8 ppm) [95], and deep-sea hydrothermal vent systems can also be enriched [35]. In recent years, the use of this metalloid in industry has led to an increased environmental presence [13,67,95,96,97]. Tellurite and tellurate are most common in the biosphere, while in the lithosphere, it is found as tellurides of gold and silver [67] and in copper ores [68,70]. Tellurite, which is the focus of this review, is the most toxic form, with levels as low as 1 µg/mL proving fatal to microorganisms [2].

### 2.2. Interactions with Microbes

The means by which tellurite exerts its toxicity is still debated; however, the strong oxidative properties [98], confirmed by an E° of 0.827 V for the TeO_3_^−2^/Te redox couple [22], are likely among the reasons. Exposure inhibits ATP production in aerobically-grown, non-resistant *E. coli* by disrupting the transmembrane proton gradient, resulting in the depletion of intracellular ATP stores [99]; in murine hepatocarcinoma cells, an 80% drop in ATP is seen in its presence [100]. Even among highly-resistant bacteria, tellurite can negatively impact ATP production, with a decrease seen in *Erythromicrobium hydrolyticum*, E4(1); *Sandaracinobacter sibiricus*, RB 16-17; *Roseococcus thiosulfatophilus*, RB3: *Erythromicrobium ezovicum*, E1; *Erythrobacter litoralis*, T4; *Shewanella fridigimarina* relative ER-Te-48; *Citromicrobium bathyomarinum*, JF1; and *Pseudoalteromonas spiralis*, Te-2-2 of 31.9%, 48.8%, 55.9%, 35.9%, 41.7%, 31.2%, 46.6%, and 87.9%, respectively [17,101]. The same detrimental effect is found with regards to protein synthesis, specifically those containing reduced thiol groups [102,103,104]. However, the majority of discoveries were made using bacteria with very low-level resistance (as low as 0.5–25 µg/mL). That being said, even highly-resistant microbes can have protein production negatively impacted, with E4(1), RB 16-17, RB3, E1, ER-Te-48, and T4 showing a decrease of 21.3%, 41.5%, 66.1%, 57.8%, 31.7%, and 19.6% respectively [17,100]. However, there are two examples of highly-resistant bacteria which break this trend. *Erythromonas ursincola*, strain KR99 and *Erythromicrobium ramosum*, E5 both show an increase in protein (66.6% and 21.2%, respectively) and ATP (15.2% and 38.9%, respectively) in the presence of tellurite [17]. The reason behind this is still a mystery, but this observation further highlights the need for more research to understand all the nuances of bacterial tellurite interactions.

Given that oxyanions of Te are so toxic, it has long been believed they cannot have a significant role in biological processes. However, Te does share similar physical and electrochemical features with its group 16 members Se and S, which can lead to its substitution in their place in proteins [105]. This has been observed in fungi, but the result was protein inactivity, suggesting erroneous inclusion [106]. The same holds true for bacteria, with tellurocysteine and telluromethionine being identified, but just like in fungi, it is a result of erroneous incorporation [107,108]. However, in 2006, anaerobic respiration using Te oxyanions was discovered [22], proving that Te plays an important positive role in the life of some microbes. Since this revelation, more isolates, along with entire communities, have been found to respire on tellurite, which is discussed later in this review.

Even though bacterial tellurite resistance/reduction has been known for almost 100 years [109], research in the field of high-level resistance is just beginning to take off. The study of microbial strategies used to achieve this type of resistance, as well as the search for more species that use Te compounds as terminal electron acceptors for dissimilatory anaerobic respiration, has just began to shed new light on how bacteria carry out the process. 

## 3. Mechanisms of Tellurite Resistance and Reduction

Many microorganisms possess mechanisms for resistance to and reduction of tellurite [17,101,110,111,112]. Even though this oxyanion is not relatively abundant globally, resistance is distributed among many different groups, from phototrophs to heterotrophs, under both aerobic and anaerobic conditions [5,9,17,18,20,22,25,101,113,114]. Resistance determinants across phylogenetically-diverse taxa have a high degree of sequence similarity, suggesting they may be elements carried over from a common ancestor, evolved for survival in an ancient, metal-rich environment [115,116], or through lateral gene transfer [117]. Alternatively, metal resistance can be encoded for plasmids, which are capable of being mobilized and transferred, conferring resistance to previously-susceptible bacteria [118]. Different mechanisms have developed to deal with the presence of the highly-toxic oxyanion, tellurite, even within closely-related species of the same genus, as seen in *Shewanella* [119], but the strategies to confer high-level resistance are just beginning to emerge.

### 3.1. Aerobic Resistance

The mechanisms of tellurite resistance/reduction under aerobic conditions are diverse. With low levels, reduction is mainly carried out through non-specific reactions. Catalases, the key enzymatic defenses against reactive oxygen species (ROS), play a part in resistance to and reduction of TeO_3_^2−^. Once in the cell, tellurite can cause the formation of intracellular ROS [120,121], resulting in significant damage. Also, because it most likely exerts its toxic effect through a high oxidizing ability, such enzymes are capable of using TeO_3_^2−^ as a substrate, reducing it to Te^0^ and minimizing the negative impact, as seen in *Staphylococcus epidermidis* [122]. In some microorganisms, such as *E. coli* and *Rhodobacter sphaeroides*, periplasmic and membrane-associated nitrate reductases reduce low levels [123,124]. Several other enzymes have been shown to aid in resistance to and reduction of small amounts of the oxyanion. The thiol:disulfide oxidoreductase of *Rhodobacter capsulatus* acts as a conduit for electrons to pass from the metalloid oxyanion and the quinone pool in the membrane, resulting in tellurite reduction [125]. In *E. coli*, exposure causes the expression of gutS [126]. The true function of this protein has yet to be determined; however, it appears to be involved in a transport of some kind, suggesting efflux of the oxyanion may be taking place. In other work, the product of the *cysK* and *cobA* genes of *Geobacillus stearothermophilis* V confer resistance in *E. coli* [127], and the dihydrolipoaminde dehydrogenase of *Aeromonas caviae* ST has NADH-dependent reducing activity [128]. Three protein fractions from *Thermus thermophiles* HB8, as well as cell-free extracts of *Mycobacterium avium*, also reduce TeO_3_^2−^, but again, the interaction is non-specific, and concentrations are low [129,130]. Proteome work in *Halomonas* sp., strain MAM, has shown that a variety of proteins are overexpressed in the presence of tellurite, but this is more of a general picture of the whole proteome response rather than a specific reaction to this oxyanion [131]. While all the mentioned enzymes reduce Te compounds, many only function in the presence of very low concentrations, and/or it is not their primary function. Plasmid-based resistance determinants have been identified, such as *kilAtelAB* from IncPα plasmid RK2 [132] and *arsR-DABC* from IncF1 plasmid R773 [133], but they do not confer very high-level resistance. The elements can be chromosomally encoded as well, such as *tehAB* from *E. coli* [96], *trgABcysK* and *telA* from *R. sphaeroides* [134], and the *tmp* gene from *Pseudomonas syringae* [135]. However, the molecular basis behind most of these systems is unclear [136]. Other strategies are employed for resistance; however, they do not involve direct enzymatic reduction. In *R. sphaeroides*, it is a means of maintaining intracellular redox poise during photosynthetic growth [137]. It has also been suggested that decreased uptake plays a role in this bacterium, as an acetate transport system is responsible for ingress [138]. Therefore, even at low concentrations (60 µg/mL), acetate competes with tellurite for entry into the cell, thereby limiting the toxicity [138]. Recently, it has been found that the transport of tellurite through this acetate permease is due to a 15–16 residue insert in *Rc*ActP2 between transmembrane helices 6 and 7. The result is a conformational change, which favors the binding and translocation of the oxyanion across the membrane [139]. A similar mechanism has also been identified in *R. capsulatus* and *E. coli*. With *R. capsulatus,* entry is a pH dependent process likely involving a phosphate transporter [140] and for *E. coli,* tellurite enters the cell through the PitA phosphate transporter [141], and mutation to the phosphate transport system [142] or deletion of PitA [139] confers a higher level of resistance. Finally, some bacteria are capable of producing volatile organic Te compounds, such as dimethyltelluride [94]. While this is a proven way of detoxification, the total amounts removed are negligible [143].

### 3.2. Anaerobic Resistance

Even less is known concerning tellurite resistance and reduction under anaerobic conditions compared to aerobic. Generally, resistance to metal(loid)s is decreased without oxygen present. In the phototrophic bacteria *R. sphaeroides*, the MIC for tellurite is much lower (4–8-fold decrease) under anoxic conditions compared to oxic [113,144]. Strain ER-Se-17L is sensitive to tellurite anaerobically, but can resist it aerobically [22], and strain CM-3 shows ~50% decrease in level of resistance in the absence of oxygen [14]. This could suggest that these compounds are of no use to microorganisms anaerobically; however, it is untrue. Certain microbes use them for energy generation through dissimilatory reduction, which will be discussed later.

### 3.3. Aerobic Anoxygenic Phototrophs and Tellurite Resistance

While many heterotrophic bacteria possess low level resistance to tellurite, it is not fair to say that high-level resistance/reduction is a common trait. The same does not hold true for AAP, especially those isolated from extreme environments [145]. With only a few exceptions, such as T4 and *Hoeflea phototrophica*, which can tolerate >1000 µg/mL tellurite [2,146], the majority of the tested AAP possessing high-level resistance were isolated from extreme environments. Over half of the taxonomically-classified AAP hail from these types of locales, and all can resist high levels of tellurite [147]. The same trend is seen among as-yet-unclassified AAP. Out of 15 isolates from the East German Creek hypersaline spring system in Manitoba, Canada, eight could grow with at least 1000 µg/mL tellurite [61], and mine tailings of the Central Mine in Nopiming Provincial Park, Manitoba, Canada, provided several AAP, all which were resistant to at least 1500 µg/mL tellurite [25]. A correlation between resistance in AAP and their habitat could be made, as extreme environments are often associated with increased metalloid levels, but any firm conclusion requires further investigation. 

AAP primarily belong to the α-Proteobacteria, with a few representatives in the β and γ subgroups. They are phylogenetically closely related to the physiological group of the purple non-sulfur bacteria (PNSB) [147], which have also received some attention due to their interactions with tellurite. In bacteria such as *R. sphaeroides*, *R. capsulatus*, *R. palustris*, and *Rhodopseudomonas viridis*, it can be used for the disposal of excess reducing power generated during photosynthesis [113]. AAP may employ a similar strategy [148]. Research has indicated that a mechanism analogous to the reoxidation of electron carriers during respiration, rather than photosynthesis, may take place aerobically in RB3 [2]. As AAP have much higher levels of resistance than their PNSB relatives, it is likely that they possess a different, or additional, mechanism(s). One proposed approach of resistance/reduction involves their highly-elevated pool of carotenoids, which have been suggested to confer protection against photooxidative damage [149]. Therefore, they make good candidates for also protecting against oxidative tellurite. Some, such as zeaxanthin and erythroxanthin sulfate, are especially good at quenching ROS [150], and make up a large portion of those found in AAP, like strains E5 and T4 possessing very high-level resistance [151]. One study has investigated the impact of tellurite on pigments in AAP [152]. The oxyanion had three main effects. First, the enhanced expression of carotenoids and/or bacteriochlorophyll (BChl) in T4, KR99, JF1, and *Erythrobacter* relative; strain EG15, supporting the idea these compounds may assist in preventing damage. Second, the influence depended strongly on culture conditions, particularly tellurite and organics concentrations. A five-fold decrease in carbon source changed the effect of the metalloid from inhibitory (42%) to stimulatory (180%) on pigment synthesis in E5. Third, the tellurite-induced expression of BChl precursors such as Mg protoporphyrin in T4, constituting the first report of tetrapyrole intermediates in AAP. It also increased the synthesis of zeaxanthin, spirilloxanthin, and β-carotene in T4, but inhibited others (e.g., bacteriorubixanthinal), and altered their characteristics by red- and blue-shifting the absorption peaks. Even with all this taken into account, investigations of resistance in AAP have received insufficient attention to elucidate details. Therefore, research into this area is of high value. Whatever the strategy may be, the product of reduction is similar to that of heterotrophic bacteria, i.e., the formation of Te^0^.

## 4. High-Level Tellurite Resistance

Unlike low-level resistance, where reduction, efflux, reduced uptake, and methylation contribute, the fate of tellurite for microbes possessing high-level resistance is always the same: reduction to elemental tellurium. With so much focus on the various low-level resistance and reduction strategies, mechanisms for increased concentrations of tellurite have received much less attention. That being said, some high-level resistance approaches have been proposed.

### 4.1. Strategies for High-Level Resistance

**Figure 5:**
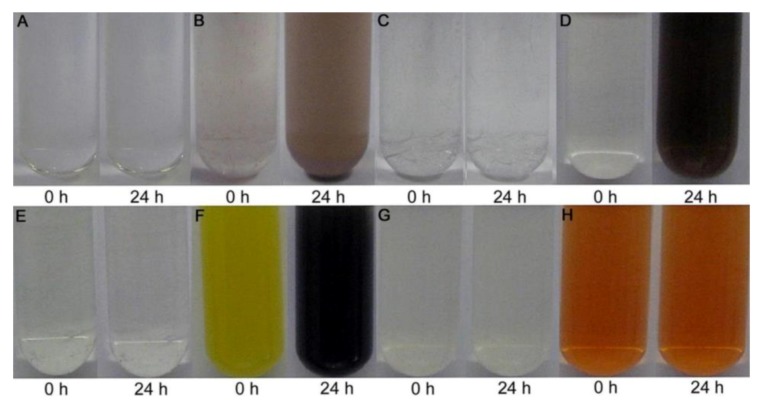
Reductase activity in cellular fractions. (**A**) Cell lysate of strain ER-Te-48 grown without prior exposure to K_2_TeO_3_. Similar results for T4, JF1, Se-1-2-red, and Te-2-2. (**B**) Strain ER-Te-48 lysate after cells exposure to K_2_TeO_3_. Initial darkening at 0 h is due to the trace presence of previously reduced K_2_TeO_3_ from exposure prior to lysis. (**C**) Strain Te-2-2 cell lysate after K_2_TeO_3_ exposure. Similar results for Se-1-2-red, T4, and JF1. (**D**) ER-Te-48 periplasmic fraction containing reductase activity following tellurite exposure. (**E**) JF1 periplasmic fraction with and without prior tellurite exposure. No reductase activity observed. Similar results for Se-1-2-red, T4, and Te-2-2. (**F**) JF1 spheroplast fraction containing reductase activity without prior exposure. T4 and Te-2-2 showed similar results. (**G**) Se-1-2-red spheroplast fraction. No reduction observed with and without prior tellurite exposure. Similar results for ER-Te-48. (**H**) T4 spheroplast lysate. No reductase activity with and without prior tellurite exposure. Similar results for JF1, Se-1-2-red, Te-2-2, and ER-Te-48. [101].

**Figure 6:**
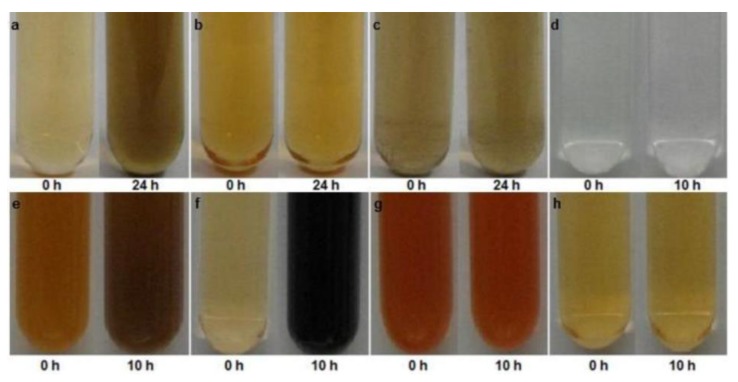
Reductase activity in cellular fractions. (**A**) Cell lysate of strain E1 grown without prior exposure to K_2_TeO_3_. Similar results were found for KR99, E5, RB3, and RB 16-17; (**B**) Lysate of strain E4(1) grown without prior exposure to K_2_TeO_3_; (**C**) Lysate of E4(1) cells grown with prior exposure to K_2_TeO_3_. Initial darkening at 0 h is due to the trace presence of previously reduced K_2_TeO_3_ from prior exposure; (**D**) Periplasmic fraction of KR99 without K_2_TeO_3_ exposure. No reductase activity observed. Similar results for E5, E4(1), E1, RB3, and RB 16-17; (**E**) Spheroplast fraction of E1 without prior K_2_TeO_3_ exposure containing reductase activity. Similar results for E5, KR99, RB3, and RB 16-17; (**F**) Spheroplast lysate of KR99 without prior K_2_TeO_3_ exposure containing reductase activity. Similar results for E5, E1, RB3, and RB 16-17; (**G**) E4(1) spheroplast fraction. No reductase activity observed with or without prior K_2_TeO_3_ exposure; (**H**) E4(1) spheroplast lysate. No reductase activity observed with or without prior K_2_TeO_3_ exposure. [17].

When it comes to specific mechanisms for resistance to and reduction of high levels of tellurite, only a very small subset of strains have been investigated. The first group studied includes five marine bacteria (Te-2-2, Se-1-2-red, T4, JF1, and ER-Te-48). This group has evolved three different strategies, all requiring de novo protein synthesis. Strain ER-Te-48 employees a periplasmic reductase for reduction, while Te-2-2, T4 and JF1 required an intact cytoplasmic membrane. Lastly, Se-1-2-red can only reduce as an undisturbed whole cell culture (Figure 5) [101]. The second group investigated involves six fresh-water aerobic anoxygenic phototrophs (E1, E4(1), E5, KR99, RB 16-17, and RB3). This group showed more consistency. All strains, with the exception of E4(1), have constitutive membrane-associated reduction [17]. E4(1), requires de novo protein preparations, as well as an undisturbed, intact cell (Figure 6) [17]. As one can see, even among this small sample group, four different strategies have emerged, making it difficult to draw any conclusions regarding a commonly-evolved approach.

### 4.2. Ter Operon

The *ter* operon is a characteristic marker found among all but one incompatibility HI2 (IncHI2) and IncHII plasmids comprised of seven genes (*ZABCDEF)* [98]. It does confer high-level resistance (minimum inhibitory concentration (MIC) of 1028 µg/mL), but the details are still under study. Work with the minimal resistance-conferring fragment (*terBCDE*) has shown that resistance can only be achieved by the expression of all four components [136]. In this case, they each play an irreplaceable role, likely involving their mutual association at the inner membrane [136]. Also, some of the individual genes from the operon actually appear to be lethal when cloned alone, making them difficult to study [153]. While the operon clearly gives resistance, it is not its main function. *TerZABCDEF* provides resistance to phages and pore-forming colicins, and it is also found in pathogenic bacteria, which have no need for tellurite resistance. Therefore, it is believed to serve some unknown function which increases fitness [136]. Also, TerD and TerE are involved in intracellular survival and the proliferation of *Yersinia pestis* in macrophages [154].

### 4.3. Tellurite Reductases

One would think that organisms which can resist and reduce these very high levels of tellurite would have some type of reductase specifically suited to the job. However, the identification of such an enzyme remained elusive for many years. At the time of writing, there are only three published examples of reductases dedicated to tellurite reduction. The first was found in 2009, when *Bacillus* sp., STG-83 was isolated from Neidasht spring in Iran, which can reduce increased levels of tellurite (~320 µg/mL) [155]. In this bacterium, the reduction of TeO_3_^2−^ is accomplished by a cytoplasmic enzyme [156]. The tellurite reductase is 197 kDa, comprised of three subunits (66, 43, and 20 kDa), functions optimally at 35 °C, pH 8.0, and has a K_m_ of 2.6 mM with a V_max_ of 5.2 µmol/min/mg protein [156]. Although it has not been confirmed, the enzyme is most likely respiratory in nature [156]. The second and third examples were both identified in 2017. One originates in the periplasm of strain ER-Te-48, isolated from a deep-ocean hydrothermal vent tube worm [111]. The enzyme, which requires *de novo* protein preparations, is 215 kDa comprised of three subunits (74, 42, and 25 kDa) in a 2:1:1 ratio. The optimum pH and temperature for activity is 8.0 and 35 °C, respectively, with tellurite reduction having a V_max_ of 5.6 µmol/min/mg protein and a K_m_ of 3.9 mM. The other comes from the aerobic anoxygenic phototroph KR99, isolated from a freshwater thermal spring of Kamchatka Island in Russia [112]. The reductase is a constitutively-expressed membrane associated enzyme with a molecular weight of 117 kDa and comprising two subunits (62 and 55 kDa) in a 1:1 ratio. Optimal activity occurs at pH 7.0 and 28 °C, with tellurite reduction having a V_max_ of 5.15 µmol/min/mg protein and a K_m_ of 3.36 mM. It should be mentioned that both these enzymes are also capable of reducing tellurate. It is unknown whether the cytoplasmic enzyme from *Bacillus* sp., STG-83 can also reduce this Te oxyanion in addition to tellurite. As one can see, it does seem that these extremely resistant microbes have specific enzymes to help carry out the task of reduction, and therefore, increased resistance. Furthermore, it appears, much like with the high-level reduction strategies discussed above, that many different approaches have evolved. These three tellurite reductases are all significantly different in nature, originating in the cytoplasm of a gram-positive bacillus, the periplasm of a gram-negative facultative anaerobe, and associated with the membrane of an aerobic anoxygenic phototroph. It is interesting to note that as different as these proteins are, all three possess a very similar V_max_, and fairly similar K_m_ values. That being said, there is currently insignificant information to attempt to draw any conclusions about the nature of tellurite reductases, with future work required to help expand on this aspect of resistance and reduction.

## 5. Anaerobic Respiration Using Tellurite

**Figure 7:**
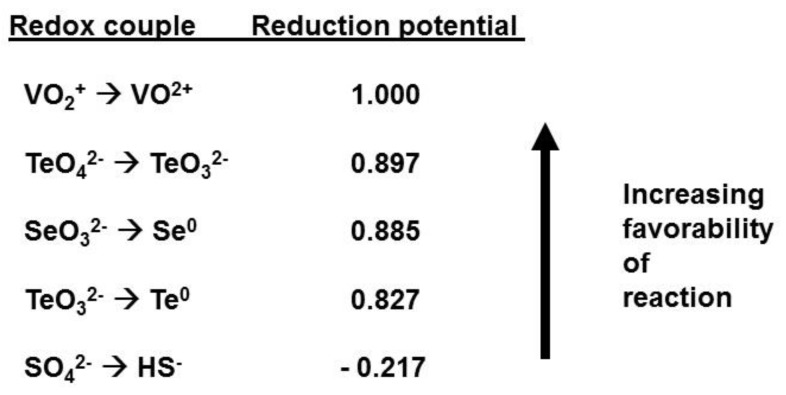
Energetics of Te, Se, and V oxyanion redox couples showing they are more favorable for anaerobic respiration than the SO^2−^/HS^−^ redox couple used by sulfate-reducing bacteria.

Microbial reduction of some metal(loid)s, such as iron (Fe), manganese (Mn), uranium (U), chromium (Cr), arsenic (As), and mercury (Hg), is well known to be coupled to the oxidation of organic or inorganic sources of energy in anaerobic respiration [157,158]. The redox couple of TeO_3_^2−^/Te (0.827 V) is more favorable for anaerobic respiration than the SO^2−^/HS^-^ redox couple (−0.217 V) utilized by sulfate, making it very favorable as a terminal electron acceptor for anaerobic respiration (Figure 7) [159]. Because sulfate-reducing bacteria can conserve energy from the latter reaction, the reduction of tellurite may also have similar potential. Also, the reduction of tellurite to Te coupled to the oxidation of lactate is highly exergonic (ΔG_fo_ = −71.3 kJ (mol electrons)^−1^) [66], providing abundant energy for growth [160]. It is likely that the factors contributing to its infrequent use are low global abundance and high toxicity. Nevertheless, respiration on other toxic oxyanions is known [157,158,161,162], indicating that toxicity to some species does not always prevent inclusion in the metabolism of others. Some bacteria, such as *Shewanella oneidensis*, MR-1 and *Bacillus* sp., STG-83, were suspected of having this capability, but this has yet to be confirmed [156,163]. Due to this, tellurite was believed to serve no beneficial biological function until 2007, when the first report of the dissimilatory anaerobic use of tellurite was published for *B. selenitireducens* and *B. beveridgei*, followed by *Sulfurospirillum barnesii* in 2009 and strain CM-3 in 2015 [14,66,164]. Based on this data, dissimilatory reduction still appeared to be a niche ability, hardly something to consider as important for survival. However, in 2016, an entire microbial community capable of using tellurite for anaerobic respiration was discovered [18]. The epibionts of tube worms inhabiting the deep-sea hydrothermal vents of the Juan de Fuca Ridge in the Pacific Ocean have evolved to utilize the highly toxic compound as a means of energy generation, and therefore, for survival. Of 107 isolates, 105 could use tellurite for growth anaerobically, showing that, around black smokers in particular, this method of respiration is not simply an ability possessed by a few select bacteria, but is an established method of energy generation for a vast diversity of microbes.

The ability to respire on tellurite does not appear to be confined to a single group. When considering the three taxonomically-classified examples, as mentioned above, two are *Bacillus* spp. (*B. beveridgei* and *B. selenitireducens*) and the remaining strain is *S. barnesii*, suggesting a limited phylogenetic range; however, considering the unclassified strains, a wider distribution is seen across several different genera (*Aquabacterium*, *Curvibacter*, *Marinobacter*, *Okibacterium*, *Pseudomonas*, *Pseudoalteromonas*, *Shewanella*, *Thalassospira*, and *Vibrio*) [18]. Based on this information, it would appear that the means of using metal(loid) oxyanions as terminal electron acceptors during anaerobic growth is a phylogenetically-widespread phenomenon, evolved by various bacterial groups, or possibly a result of lateral gene transfer between members of multiple genera [117]. One could also speculate it is a genetic element left over from ancient ancestors who developed the skill to survive on a primordial, metal-rich earth [165]. As species and genera diverged over time, the genes for the process remained.

## 6. Potential Role in Bioremediation and Biometallurgy

In the past, accidents and poor operating procedures have resulted in the release of vast quantities of toxic sludge [166,167,168], adding urgency to the need for remediation. Microorganisms that are capable of reducing and removing tellurite may provide ecologically-friendly approaches for clean-up efforts, as well as for the possible biorecovery of elemental Te for use in industry.

### 6.1. Bioremediation

Increased concentrations of tellurite in the environment have led to the search for removal methods which will not enhance the original pollution issue. The neutralization of toxic oxyanions using chemicals and resins has been employed [169,170]; however, they present problems related to their high cost and the inherent fact that they proliferate the release of xenobiotic compounds themselves. More interest in environmentally-friendly, ‘greener’ methods of dealing with tellurite has arisen, with biological approaches appearing to be the ideal way of cleaning up pollutants [12,171]. Microbes with the ability to reduce oxyanions from highly toxic states to less toxic elemental forms as a means to remediate contaminated sites have been in the spotlight. The removal of xenobiotics, metals, and radioactive compounds through bioremediation has been explored [31,171,172,173,174,175,176,177], but little has been proposed regarding tellurite treatments. The use of microbial communities, i.e., *Pseudomonas mendocina*, strain MCM B-180 [177], and *Pseudoalteromonas* sp., EPR3 [40], has been investigated [12,178,179]. The most effective bacterium for tellurite removal is currently *P. mendocina*, strain MCM B-180, performing optimally at a tellurite concentration of 10 µg/mL taking 72 h to remove 100 µg/mL (1.4 mg/L/h) [180]. As one can see, these microbes do in fact help remediate tellurite; however, initial concentrations of the oxyanion are low, and days are required before significant removal takes place. Therefore, the currently-proposed strains leave much room for improvement. Recently, bacteria possessing greater resistance levels with the ability for faster reduction have shown to be more effective and efficient [181]. Under aerobic conditions, strains KR99, E5, AV-Te-18 and ER-V-8 removed 203, 244, 98, and 93 µg/mL tellurite (4.2, 5.1, 2.1, and 2.0 mg/L/h), respectively, after 48 h, which is a significantly higher level than that reported for *P. mendocina* (Table 1). In the case of anaerobic conditions, the bioremediation of tellurite has not been significantly investigated; however, strains ER-V-8 and AV-Te-18 are both capable of removing 10 µg/mL tellurite, the same concentration *P. mendocina* functions optimally at aerobically, under anoxic conditions, with ER-V-8 taking 48 h (0.2 mg/L/h) and AV-Te-18 only requiring 24 h (0.4 mg/L/h) (Table 1). For strain AV-Te-18, the total removal can be as high as 51 µg/mL after 5 days. Strains such as these may prove to provide a feasible means of bioremediation, but this is yet to be determined. 

### 6.2. Biometallurgy

**Figure 8:**
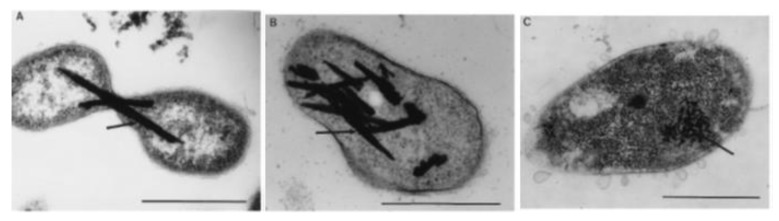
Electron microscopy of ultrathin sections. Shown are the intracellular localizations of presumed tellurium (indicated by arrows) as a product of tellurite reduction. (**A**) Two daughter cells of E5 with tellurium crystals apparently interfering with cell division. (**B**) In T4, tellurium crystals sometimes occupy as much as 20% to 30% of the cell volume. (**C**) RB3 accumulates relatively small Te crystals similar to Te deposits observed in *E. coli* or *Rhodobacter* species. Scale Bars, 0.5 mm. [15].

Another area of great interest involving biological transformations of metal(loid)s is biometallurgy [182]. This involves the use of microbes for retrieval, or ‘mining’, of the desired elements from ores. Currently, the costs associated with classic techniques of mining are increasing, and efficiency is declining. Therefore, the use of biotic methods for recovery is gaining popularity [183,184]. Some approaches are already employed for several applications. *Thiobacillus ferrooxidans* and *T. thiooxidans*, which are capable of Fe- and S-oxidation, respectively, have been used to disrupt the FeS_2_ (pyrite) matrix of ores containing metals of interest. The biooxidation is 10^6^ times faster than abiotic oxidation [185], highlighting the appeal. The process also results in the release of solubilized Te and V [186], providing a further source of contamination. With Te, for example, a biological approach to recovery is highly sought after [187]. The element is quite scarce in the Earth’s crust, and the current methods for production and recycling are inefficient, with up to 90% of Te being lost [40,188]. Currently, separation and purification is highly complex. One method used in the United States is a week-long process which involves reducing tellurium dioxide (TeO_2_) to elemental tellurium (Te^0^) through high-pressure and high-temperature autoclaving in concentrated hydrochloric acid and sulfur dioxide [40]. Since this is tedious and involves dangerous chemicals, an alternative purification would be highly desirable from a safety and environmental perspective. Projections indicate that recycling of Te could virtually eliminate problems associated with its scarcity [189], further fueling the search for bacteria with the potential for microbial reclamation of the highly critical and rare element. Not only would it provide a means of recovery, but also bioremediation, aiding in removal from the biosphere. As the end-product of tellurite reduction by these highly resistant microbes is pure elemental Te crystals, internalized in the cell (Figure 8), they appear to have the ability to aid in the recovery of this desirable metalloid, potentially providing an ecologically-friendly means of recycling and recovery.

## 7. Summary and Perspectives

As one can see, while bacterial tellurite resistance has been known of for almost a century [109], numerous questions still remain. It is only recently that significant discoveries are being made with regards to the physiological effect of the oxyanion, the enzymes involved in resistance/reduction, and the fact that tellurite is actually a biologically-relevant compound. Much still remains a mystery where very high-level resistance is concerned, and while these recent findings have begun to shed light on the subject, we have only just started to scratch the surface. As concern surrounding Te contamination has increased in recent years, further study on microbe–tellurite interactions is needed, especially involving those bacteria which may provide a means to alleviate environmental contamination. Obviously, bacteria with the ability to resist and reduce tellurite are of great interest for many different fields of applied science. Future research has the chance to yield some important discoveries in bioremediation, bioreclamation, biometallurgy, enzymology, microbial ecology, microbial physiology, and taxonomy. One can only imagine the new discoveries that lie ahead. 

## Figures and Tables

**Table 1:** Amount of tellurite, removed (mg/L/h) under aerobic and anaerobic conditions for *P. mendocina* strain MCM B-180, *Erythromonas ursincola* KR99, *Erythromicrobium ramosum* E5, AV-Te-18, and ER-V-8.

Strain	Aerobic	Anaerobic
*P. mendocina* MCM B-180	1.4	NA
*Erythomonas ursincola* KR99	4.2	NA
*Erythromicrobium ramosum* E5	5.1	NA
AV-Te-18	2.1	0.4
ER-V-8	2.0	0.2
